# Effects of intervention design on engagement and outcomes in digital self-help for insomnia – factorial RCT

**DOI:** 10.1038/s41746-025-01839-0

**Published:** 2025-07-08

**Authors:** Amira Hentati, Nils Hentati Isacsson, Ann Rosén, Susanna Jernelöv, Viktor Kaldo, Brjánn Ljótsson, Erik Forsell, Nils Lindefors, Martin Kraepelien

**Affiliations:** 1 Centre for Psychiatry Research, Department of Clinical Neuroscience, Karolinska Institutet, & Stockholm Health Care Services, Region Stockholm, Stockholm, Sweden; 2 Division of Psychology, Department of Clinical Neuroscience, Karolinska Institutet, Stockholm, Sweden; 3 Department of Psychology, Faculty of Health and Life Sciences, Linnaeus University, Växjö, Sweden

**Keywords:** Outcomes research, Psychiatric disorders, Health services, Randomized controlled trials

## Abstract

Digital self-help can improve access to mental health care, but poor engagement limits effectiveness. This single-blind 2 x 2 x 2 factorial randomized controlled trial examined whether an optimized graphical user interface (GUI), automated reminders (AR), and an adaptive treatment strategy (ATS) improved engagement and outcomes in a digital self-help insomnia intervention. Adults (*N* = 447) with moderate to severe insomnia were randomized to combinations of the factors. The GUI improved self-rated engagement, sleep log activity, login frequency, and usability. AR increased sleep log activity and logins, while ATS improved satisfaction. All three combined significantly improved insomnia symptoms (*d* = 0.50). No severe adverse effects were reported. Clinicians spent 13.74 min on average on the ATS. Statistical analyses included linear and multilevel regression. Factors were effect coded. Intervention design can enhance engagement and outcomes, requiring minimal clinician time. Pre-registered 2023-04-11 (ClinicalTrials.gov, NCT05826002). Funded by the Swedish Ministry of Health and Social Affairs, grant number: S2018/03855/FS.

## Introduction

Cognitive Behavioral Therapy (CBT) has proven to be an effective treatment for a range of mental health problems^1^. Unfortunately, the availability of interventions derived from CBT is limited due to a shortage of qualified clinicians and unequal access to care^2,3^. Digital self-help interventions, where patients work independently with a prescribed digital intervention, could facilitate dissemination of treatment^4^. If interventions can be delivered without clinician guidance, fewer resources are needed, increasing reach. However, challenges with treatment engagement have been observed in self-help interventions and are associated with reduced treatment effects^4,5^.

Engagement has been defined by two key dimensions: 1) the extent of usage, and 2) the subjective experience of usage^6^. In behavioral science, the focus has predominantly been on the first dimension, emphasizing aspects such as the frequency, duration, and depth of engagement with treatment interventions^6^. Given the close association between engagement and effectiveness of digital interventions^7^, understanding and measuring engagement has been a longstanding area of interest^6^. To achieve this, both self-rated questionnaires and objective measures are widely used, capturing different dimensions of engagement^6^.

To address the growing demand for treatment, achieving satisfactory engagement and outcomes in digital self-help interventions is an important step. Designing the intervention thoughtfully has been proposed as a key aspect in enhancing engagement^8^. Among the features considered potentially vital are intuitive interface design and easy navigation, reminder systems to prompt engagement, and procedures to identify and provide additional support to patients at risk of not sufficiently benefiting from treatment.

Interface design is suggested to influence engagement with digital interventions^9^. An optimized graphical user interface (GUI), characterized by user-friendly features that simplify interaction, is often highlighted as key in improving user engagement^10,11^. The idea is that an optimized GUI will facilitate engagement through intuitive navigation of the material, and by reducing the risk of technical difficulties^10^. However, there is a lack of studies that investigate the causal relationship between GUI design and engagement. In a randomized controlled trial conducted by our research group, participants facing emotional or practical challenges during the COVID-19 pandemic demonstrated greater engagement with a self-guided problem-solving intervention when using an optimized GUI compared to a basic one^12^. This study had some limitations, including a brief intervention period and a lack of clinical criteria for participation.

Reminders represent another strategy suggested to boost engagement with digital interventions^13,14^. Automated reminders – reminders that are not human activated – have been used in various digital interventions as a strategy to prompt users to engage with the treatment material^13^. While some studies have indicated that reminders have a positive influence on engagement and outcomes^13,15,16^, other studies have not been able to confirm this^17^. In self-guided interventions, reminders could perhaps function as a substitute to reminders coming from the clinician in guided interventions.

An adaptive treatment strategy is a form of measurement-based care^18^, and involves early assessment during treatment to identify individuals who may be at risk of not sufficiently benefiting from the intervention, followed by tailored adjustments to meet specific needs^19^. Previous research has demonstrated that an adaptive treatment strategy can reduce treatment failures and enhance outcomes in digital therapist-guided CBT for insomnia (CBT-I)^19^. One of the key benefits of adaptive treatment strategies is that they intensify support only for those who need it, making it a more cost-effective approach than providing tailored adjustments to everyone. Given that digital self-help interventions are typically designed for independent use, it is plausible that they could also benefit from an adaptive treatment strategy, particularly by integrating therapist support for participants who struggle with engagement. However, this has yet to be tested.

Insomnia is a widespread public health issue, associated with substantial costs both for individuals and society^20^. As outlined in international guidelines, CBT-I is the first-line treatment and has been shown to be highly effective^21^. Given the high prevalence of insomnia and the well-established efficiency of CBT-I, it is an ideal condition for optimizing digital self-help interventions, prompting this trial to enhance engagement and outcomes in a digital program based on CBT-I components.

The aim of this study was to investigate whether engagement with and outcomes of a digital self-help intervention for insomnia could be optimized through the inclusion of additional intervention features: 1) an optimized GUI, 2) automated reminders, and 3) an adaptive treatment strategy. The core version of the intervention, called FastAsleep, was developed and evaluated in a previous feasibility study^22^.

The specific research questions were: Are there main or interaction effects of the three features on treatment engagement, insomnia symptoms, usability, treatment satisfaction or credibility in a digital self-help intervention for insomnia during the active treatment period and at long-term follow-up up to one year after treatment? How much time do clinicians spend on these additional features? Are any negative effects evenly distributed across the features?

In a forthcoming publication, we will evaluate and report on the overall intervention, including secondary outcomes such as quality of life, daily functioning, and symptoms of depression and anxiety, as well as the broader impact of the adaptive treatment strategy. This distinction ensures that the scope and findings of the current study remain clear and focused.

## Results

**Fig. 1:**
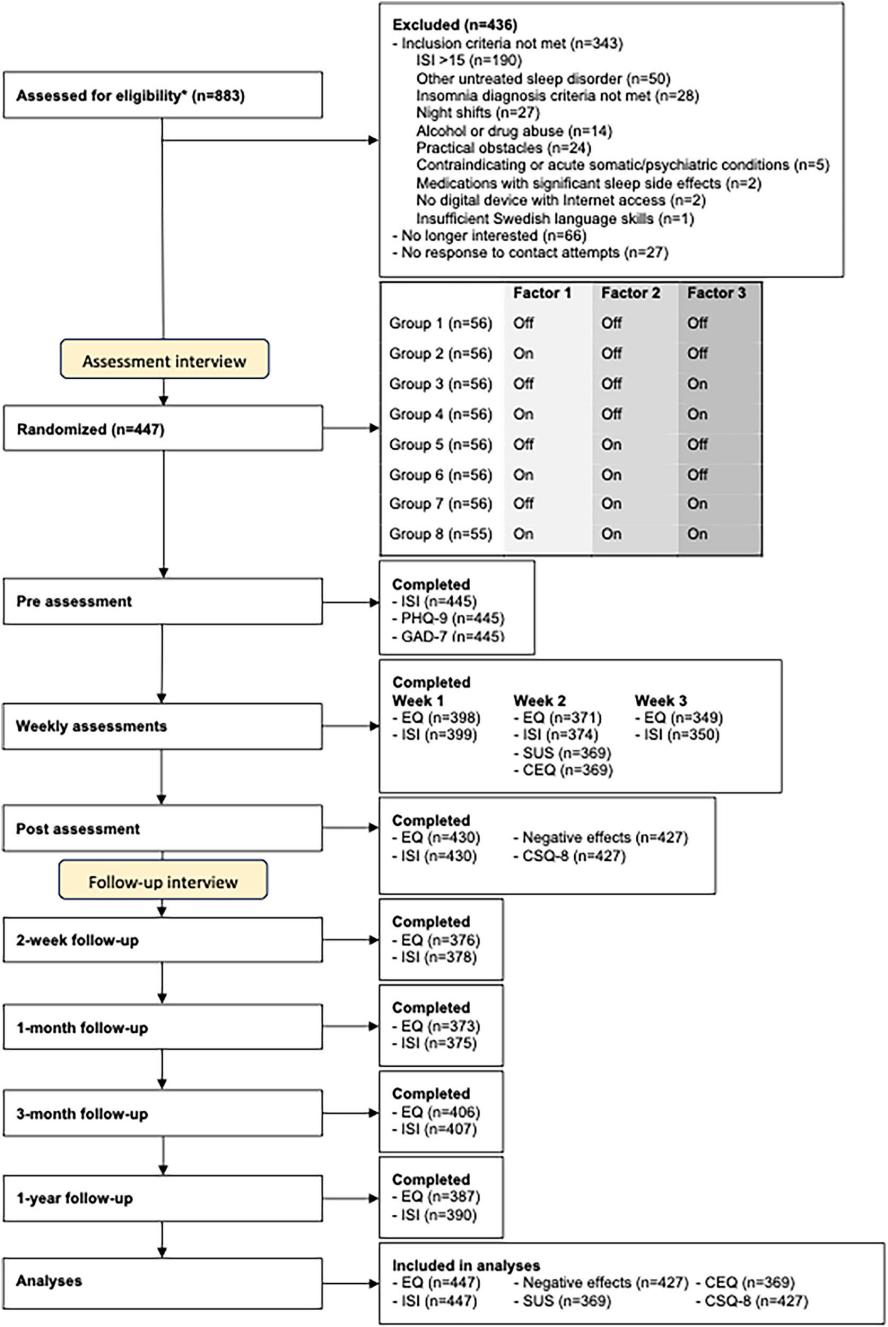
Trial profile. *The Insomnia Severity Index measured at this point served as baseline data for participants subsequently included in the study; EQ Engagement Questionnaire, ISI\ Insomnia Severity Index, PHQ-9 Patient Health Questionnaire-9, GAD-7 Generalized Anxiety Disorder-7, SUS System Usability Scale, CEQ Credibility/Expectancy Questionnaire, CSQ-8 Client Satisfaction Questionnaire-8.

A total of 883 individuals completed a registration to the study, of which 447 (51%) were included and randomized, see Fig. 1. Due to a high number of applicants, a slightly larger number of participants than planned was included. For each factor in the experiment, 223 participants were in the ‘on’ condition, and 224 in the ‘off’ condition. Among those who were allocated to the adaptive treatment strategy factor (*n* = 223), a total of 123 participants (55%) were assessed as at risk of not sufficiently benefiting from treatment and were thus offered individualized support.

**Fig. 2:**
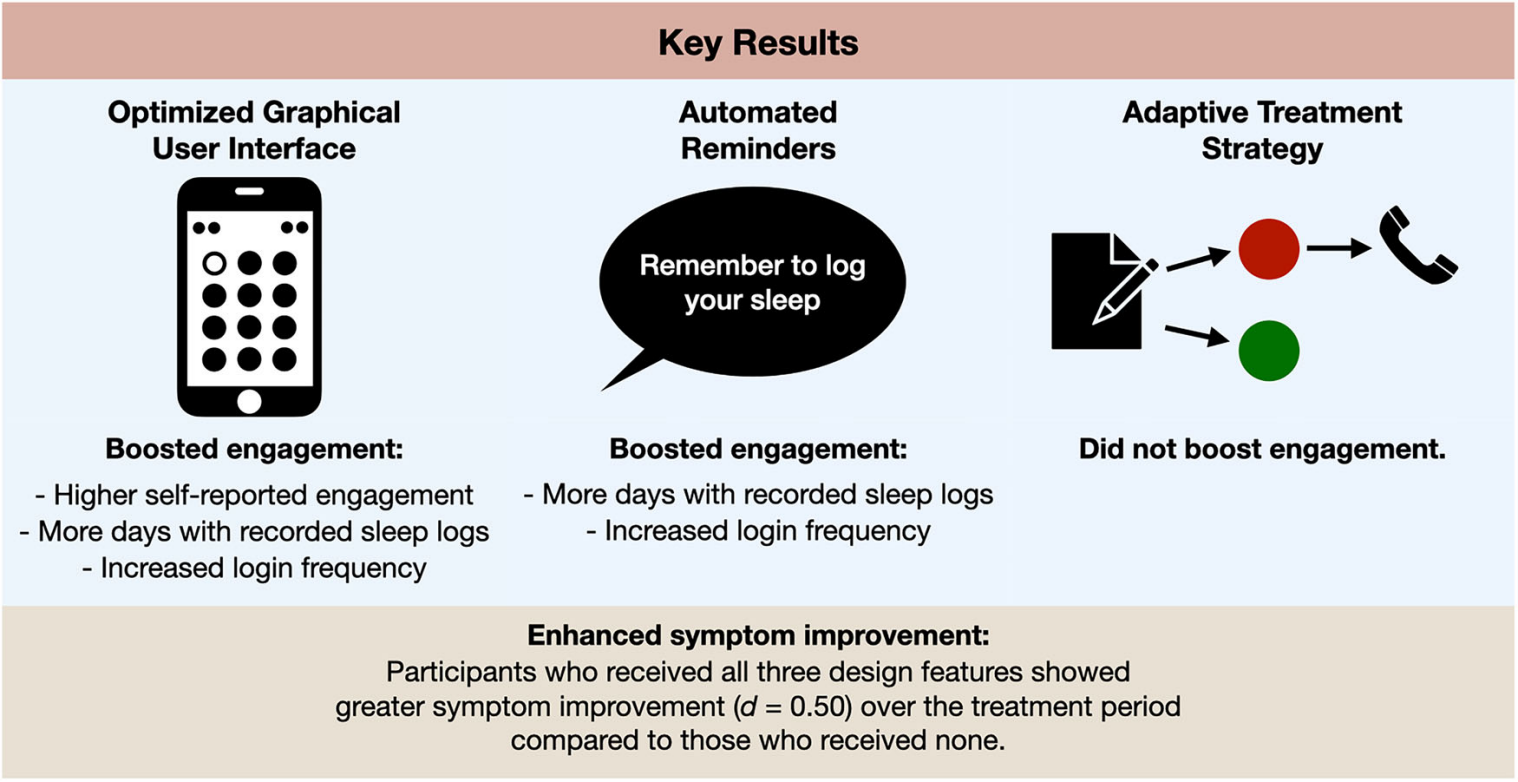
Key results. Visualization of key results on treatment engagement and outcomes of the factors.

The majority of participants were female and had a university education. The mean score for insomnia symptoms indicated moderate severity, while depressive symptoms also reflected moderate severity, and anxiety symptoms were rated as mild. See Table 1 for complete baseline characteristics of the sample. For descriptive statistics for all outcome measures, see Table 2. For an overview of results, see Fig. 2.

**Table 1:** Baseline characteristics

	Total sample (*n* = 447)*
**Female**, ***n*** **(%)**	388 (87%)
**Age, mean (SD) [range]**	54 (12) [21–85]
**University education**, ***n*** **(%)**	321 (72%)
**Long-term sick leave**, ***n*** **(%)**	47 (11%)
**Not working full time partly or completely due to sleep disorder**, ***n*** **(%)**	75 (17%)
**Baseline insomnia symptoms (ISI score), mean (SD) [range]**	19.51 (3.11) [15–28]
**Years with troubled sleep, mean (SD) [range]**	16.71 (13.11) [0-63]
**Baseline depressive symptoms (PHQ-9), mean (SD) [range]**	10.28 (4.78) [1–25]
**Baseline anxiety symptoms (GAD-7), mean (SD) [range]**	6.83 (4.99) [0-21]

*For PHQ-9 and GAD-7, *n* = 445, *ISI* Insomnia Severity Index, *PHQ-9* Patient Health Questionnaire-9, *GAD-7* Generalized Anxiety Disorder-7, *SD* Standard deviation.

**Table 2:** Descriptive data on primary and secondary outcome measures for the entire group and per factor

	Entire Group	Optimized Graphical User Interface		Automated Reminders		Adaptive Treatment Strategy	
		Off	On	Off	On	Off	On
**EQ treatment period (1 week to post) (scoring 0-21), mean [95% CI] (SD)**	*n* = 447 12.80 [12.23, 13.37] (6.08)	*n* = 224 11.79 [10.95, 12.63] (6.38)	*n* = 223 13.82 [13.09, 14.56] (5.59)	*n* = 224 12.64 [11.84, 13.43] (6.05)	*n* = 223 12.97 [12.16, 13.78] (6.12)	*n* = 224 12.46 [11.63, 13.28] (6.26)	*n* = 223 13.15 [12.37, 13.93] (5.89)
**EQ short-term follow-up period (post to three-month follow-up) (scoring 0-21), mean [95% CI] (SD)**	*n* = 447 9.04 [8.54, 9.53] (5.37)	*n* = 224 8.39 [7.71, 9.06] (5.11)	*n* = 223 9.69 [8.96, 10.42] (5.55)	*n* = 224 9.20 [8.50, 9.90] (5.33)	*n* = 223 8.87 [8.16, 9.58] (5.41)	*n* = 224 8.69 [8.01, 9.36] (5.11)	*n* = 223 9.39 [8.65, 10.13] (5.60)
**EQ one-year follow-up (scoring 0-21), mean [95% CI] (SD)**	*n* = 447 7.70 [7.18, 8.21] (5.56)	*n* = 224 7.48 [6.77, 8.20] (5.42)	*n* = 223 7.91 [7.16, 8.66] (5.69)	*n* = 224 7.83 [7.12, 8.54] (5.45)	*n* = 223 7.57 [6.82, 8.31] (5.67)	*n* = 224 7.40 [6.66, 8.13] (5.58)	*n* = 223 8.00 [7.27, 8.73] (5.54)
**Number of days with recorded sleep logs during treatment (0-27 registrations), mean [95% CI] (SD)**	*n* = 447 19.32 [18.35, 20.30] (10.48)	*n* = 224 17.05 [15.54, 18.55] (11.40)	*n* = 223 21.61 [20.44, 22.79] (8.92)	*n* = 224 17.99 [16.54, 19.43] (10.97)	*n* = 223 20.67 [19.38, 21.96] (9.80)	*n* = 224 18.89 [17.46, 20.32] (10.85)	*n* = 223 19.76 [18.43, 21.09] (10.10)
**Number of logins during treatment, mean [95% CI] (SD)**	*n* = 447 22.06 [20.92, 23.21] (12.31)	*n* = 224 20.39 [18.62, 22.15] (13.40)	*n* = 223 23.74 [22.31, 25.18] (10.88)	*n* = 224 20.07 [18.43, 21.72] (12.49)	*n* = 223 24.06 [22.50, 25.62] (11.81)	*n* = 224 21.66 [20.08, 23.24] (12.01)	*n* = 223 22.47 [20.80, 24.13](12.61)
**ISI baseline (scoring 0-28), mean [95% CI] (SD)**	*n* = 447 19.51 [19.22, 19.80] (3.11)	*n* = 224 19.56 [19.16, 19.97] (3.07)	*n* = 223 19.46 [19.05, 19.88] (3.15)	*n* = 224 19.46 [19.06, 19.87] (3.06)	*n* = 223 19.56 [19.15, 19.98] (3.16)	*n* = 224 19.75 [19.33, 20.17] (3.19)	n = 223 19.27 [18.87, 19.67] (3.01)
**ISI post-treatment (scoring 0-28), mean [95% CI] (SD)**	*n* = 430 11.34 [10.82, 11.86] (5.52)	*n* = 213 11.57 [10.81, 12.34] (5.65)	*n* = 217 11.11 [10.39, 11.83] (5.40)	*n* = 217 11.70 [10.93, 12.48](5.79)	*n* = 213 10.97 [10.27, 11.68] (5.23)	*n* = 220 11.72 [10.97, 12.47] (5.67)	*n* = 210 10.94 [10.22, 11.67] (5.35)
**ISI three-month follow-up (scoring 0-28), mean [95% CI] (SD)**	*n* = 407 11.35 [10.80, 11.90] (5.66)	*n* = 202 11.58 [10.80, 12.37] (5.63)	*n* = 205 11.12 [10.34, 11.91] (5.68)	*n* = 208 11.57 [10.76, 12.38] (5.91)	*n* = 199 11.13 [10.37, 11.88] (5.38)	*n* = 206 11.77 [10.96, 12.58] (5.91)	*n* = 201 10.93 [10.18, 11.67] (5.36)
**ISI one-year follow-up (scoring 0–28), mean [95% CI] (SD)**	*n* = 390 11.76 [11.19, 12.32] (5.68)	*n* = 189 11.79 [10.96, 12.62] (5.78)	*n* = 201 11.73 [10.95, 12.50] (5.60)	*n* = 201 11.91 [11.09, 12.73] (5.87)	*n* = 189 11.59 [10.81, 12.38] (5.48)	*n* = 196 11.90 [11.07, 12.73] (5.46)	*n* = 194 11.61 [10.84, 12.39] (5.46)
**System Usability Scale (scoring 0–100), mean [95% CI] (SD)**	*n* = 369 74.00 [72.12, 75.87] (18.31)	*n* = 169 69.97 [67.00, 72.94] (19.59)	*n* = 200 77.40 [75.10, 79.70] (16.46)	*n* = 180 74.06 [71.46, 76.65] (17.64)	*n* = 189 73.94 [71.22, 76.67] (18.98)	*n* = 182 74.23 [71.49, 76.98] (18.78)	*n* = 187 73.77 [71.19, 76.35] (17.90)
**Credibility/Expectancy Questionnaire (scoring 0–50), mean [95% CI] (SD)**	*n* = 369 33.26 [32.21, 34.31] (10.25)	*n* = 169 32.88 [31.29, 34.48] (10.49)	*n* = 200 33.58 [32.18, 34.98] (10.06)	*n* = 180 33.49 [32.01, 34.97] (10.09)	*n* = 189 33.04 [31.55,34.54] (10.43)	*n* = 182 32.65 [31.08, 34.22] (10.72)	*n* = 187 33.86 [32.45, 35.27] (9.77)
**Client Satisfaction Questionnaire-8 (scoring 8–32), mean [95% CI] (SD)**	*n* = 427 22.69 [22.19, 23.19] (5.25)	*n* = 211 22.37 [21.65, 23.08] (5.30)	*n* = 216 23.01 [22.31, 23.70] (5.20)	*n* = 216 22.68 [21.98, 23.37] (5.21)	*n* = 211 22.70 [21.98, 23.42] (5.31)	*n* = 218 21.76 [21.04, 22.47] (5.35)	*n* = 209 23.66 [22.98, 24.34] (4.98)

*EQ* Engagement Questionnaire, *ISI* Insomnia Severity Index, *CI* Confidence interval, *SD* Standard deviation.

### Missing data

On the EQ and ISI, there were 4% missing data at post-treatment, 9% missing data at three-month follow-up, and 13% missing data at one-year follow-up.

### Engagement questionnaire

The regression models for EQ (see Table 3) showed a main effect of the optimized GUI during the treatment period and short-term follow-up period. During the treatment period, engagement ratings were 2.04 points higher, corresponding to an effect size of *d* = 0.34 (95% CI [0.15, 0.53]). During the short-term follow-up period, they were 1.30 points higher, *d* = 0.24 (95% CI [0.06, 0.43]).

**Table 3:** Effects of the factors on the Engagement Questionnaire

	EQ treatment period (week 1 to post)				EQ short-term follow-up period (post to three-month follow-up)				EQ one-year follow-up			
	Estimate [95% CI]	b_corr_ [95% CI]	SE	*p*	Estimate [95% CI]	b_corr_ [95% CI]	SE	*p*	Estimate [95% CI]	b_corr_ [95% CI]	SE	*p*
**Intercept**	12.81 [12.25, 13.36]	NA	0.28	<0.0001*	9.04 [8.54, 9.53]	NA	0.25	<0.0001*	7.70 [7.18, 8.21]	NA	0.26	<0.0001*
**Optimized GUI**	2.04 [0.93, 3.16]	2.04 [0.93, 3.16]	0.57	0.0004*	1.30 [0.31, 2.30]	1.30 [0.31, 2.30]	0.51	0.0102*	0.43 [−0.61, 1.46]	0.43 [−0.61, 1.46]	0.53	0.4164
**Automated reminders**	0.34 [−0.77, 1.46]	0.34 [−0.77, 1.46]	0.57	0.5463	−0.32 [−1.32, 0.67]	−0.32 [−1.32, 0.67]	0.51	0.5215	−0.26 [−1.29, 0.78]	−0.26 [−1.29, 0.78]	0.53	0.6233
**ATS**	0.70 [−0.42, 1.81]	0.70 [−0.42, 1.81]	0.57	0.2199	0.71 [−0.29, 1.70]	0.71 [−0.29, 1.70]	0.51	0.1636	0.60 [−0.44, 1.63]	0.60 [−0.44, 1.63]	0.53	0.2568
**Optimized GUI× Automated reminders**	1.73 [−0.51, 3.96]	0.87 [−0.26, 1.98]	1.14	0.1294	0.73 [−1.26, 2.71]	0.37 [−0.63, 1.36]	1.01	0.4729	−0.09 [−2.16, 1.98]	−0.05 [−1.08, 0.99]	1.05	0.9325
**Optimized GUI×ATS**	1.00 [−1.23, 3.23]	0.50 [−0.62, −1.62]	1.14	0.3800	−0.10 [−2.08, 1.89]	−0.05 [−1.04, 0.95]	1.01	0.9251	1.41 [−0.66, 3.48]	0.71 [−0.33, 1.74]	1.05	0.1813
**Automated reminder ×ATS**	−0.47 [−2.70, 1.77]	−0.24 [−1.35, 0.89]	1.14	0.6820	0.73 [−1.25, 2.72]	0.37 [−0.63, 1.36]	1.01	0.4693	−0.82 [−2.89, 1.25]	−0.41 [−1.45, 0.63]	1.05	0.4361
**Optimized GUI ×Automated reminder ×ATS**	−0.73 [−5.19, 3.74]	−0.18 [−1.30, 0.94]	2.27	0.7494	−1.62 [−5.59, 2.36]	−0.41 [−1.40, 0.59]	2.02	0.4238	−1.64 [−5.78, 2.50]	−0.41 [−1.45, 0.63]	2.11	0.4361

*Statistically significant effect, *EQ* Engagement Questionnaire, *CI* Confidence interval, *b*_corr_ Corrected estimate, see Methods for details, *SE* Standard error, *NA* Not applicable, *Optimized GUI* Optimized graphical user interface, *ATS* Adaptive treatment strategy.

### Number of days with recorded sleep logs

There was a statically significant main effect of the optimized GUI on the number of days with recorded sleep logs during treatment, with b = 4.58 more days (95% CI [2.69, 6.47], *p* < 0.0001) than others. For automated reminders, the increase was also statistically significant but smaller, with b = 2.69 more days (95% CI [0.80, 4.59], *p* = 0.0054). There was no statistically significant main effect of the adaptive treatment strategy on the number of days with recorded sleep logs (b = 0.88, 95% CI [−1.01, 2.77], *p* = 0.3600). See Supplementary Table 1 for details.

The number of days with recorded sleep logs during treatment correlated statistically significantly with the average total EQ score over the treatment period (r(445) = 0.82, *p* < 0.0001).

### Number of Logins

There was a statistically significant main effect of the optimized GUI on number of logins during treatment, with an increase of b = 3.37 more logins (95% CI [1.13, 5.61], *p* = 0.0033) compared to others. A similar effect was observed for automated reminders, which resulted in b = 4.00 additional logins (95% CI [1.76, 6.24], *p* = 0.0005). There was also a statistically significant interaction effect of the optimized GUI × automated reminders on the number of logins, b_corr_ = 2.25 logins (95% CI [0.01, 4.49], *p* = 0.0496). There was no statistically significant main effect of the adaptive treatment strategy on the number of logins (b = 0.82, 95% CI [−1.42, 3.07], *p* = 0.4697). For details, see Supplementary Table 1.

### Insomnia Symptoms

The regression model for the ISI (see complete details in Table 4) showed that during treatment (Phase 1), insomnia symptoms improved statistically significantly for the entire group by 8.45 points, *d* = 1.60 (95% CI [−1.69, −1.52]). However, during the follow-up period (Phase 2), there was a statistically significant but slight deterioration of 1.53 points. The total change from baseline to one-year follow-up, including both phases, resulted in a statistically significant improvement with an effect size of *d* = 1.31 (95% CI [−1.49, −1.14]).

**Table 4:** Effects of the factors and time on the Insomnia Severity Index

	Insomnia Severity Index			
	Estimate [95% CI]	b_corr_ [95% CI]	SE	*p*
**Intercept**	10.73 [10.31, 11.16]	NA	0.22	<0.0001*
***Fixed effects***				
**Optimized GUI (post-treatment)**	−0.72 [−1.57, 0.12]	−0.72 [−1.57, 0.12]	0.43	0.0941
**Automated reminders (post-treatment)**	−0.61 [−1.45, 0.24]	−0.61 [−1.45, 0.24]	0.43	0.1610
**ATS (post-treatment)**	−0.50 [−1.35, 0.34]	−0.50 [−1.35, 0.34]	0.43	0.2433
**Phase 1**	−1.69 [−1.78, −1.61]	−1.69 [−1.78, −1.61]	0.04	<0.0001*
**Phase 2**	0.03 [0.02, 0.04]	0.03 [0.02, 0.04]	0.00	<0.0001*
**Optimized GUI×Automated reminders**	0.89 [−0.80, 2.59]	0.45 [−0.40, 1.30]	0.86	0.3008
**Optimized GUI×ATS**	−0.74 [−2.44, 0.95]	−0.37 [−1.22, 0.48]	0.86	0.3896
**Automated reminders×ATS**	−0.16 [−1.85, 1.54]	−0.08 [−0.93, 0.77]	0.86	0.8550
**Optimized GUI×Phase 1**	−0.02 [−0.19, 0.15]	−0.02 [−0.19, 0.15]	0.09	0.7850
**Optimized GUI×Phase 2**	0.01 [−0.01, 0.03]	0.01 [−0.01, 0.03]	0.01	0.2190
**Automated reminders×Phase 1**	−0.16 [−0.33, 0.01]	−0.16 [−0.33, 0.01]	0.09	0.0683
**Automated reminders×Phase 2**	0.01 [−0.01, 0.03]	0.01 [−0.01, 0.03]	0.01	0.3751
**ATS×Phase 1**	−0.07 [−0.24, 0.10]	−0.07 [−0.24, 0.10]	0.09	0.4154
**ATS×Phase 2**	0.01 [−0.01, 0.02]	0.01 [−0.01, 0.02]	0.01	0.5634
**Optimized GUI×Automated reminders×ATS**	−3.36 [−6.75, 0.03]	−0.84 [−1.69, 0.01]	1.73	0.0525
**Optimized GUI×Automated reminders×Phase 1**	0.07 [−0.27, 0.41]	0.04 [−0.14, 0.21]	0.17	0.6903
**Optimized GUI×Automated reminders×Phase 2**	−0.04 [−0.07, 0.00]	−0.02 [−0.04, 0.00]	0.02	0.0391*
**Optimized GUI×ATS×Phase 1**	−0.08 [−0.42, 0.26]	−0.04 [−0.21, 0.13]	0.17	0.6288
**Optimized GUI×ATS×Phase 2**	0.01 [−0.03, 0.04]	0.01 [−0.02, 0.02]	0.02	0.6266
**Automated reminders×ATS×Phase 1**	−0.26 [−0.60, 0.08]	−0.13 [−0.30, 0.04]	0.17	0.1325
**Automated reminders×ATS×Phase 2**	0.03 [0.00, 0.07]	0.02 [0.00, 0.04]	0.02	0.0896
**Optimized GUI×Automated reminders×ATS×Phase 1**	−1.10 [−1.78, −0.42]	−0.28 [−0.45, −0.11]	0.35	0.0017*
**Optimized GUI×Automated reminders×ATS×Phase 2**	0.03 [−0.04, 0.10]	0.01 [−0.01, 0.03]	0.04	0.4421
***Random effects***				
**Variance intercept**	17.81			
**Variance slope Phase 1**	0.46			
**Variance slope Phase 2**	0.00			
**Residual**	9.62			

*Statistically significant effect, *CI* Confidence interval, *b*_corr_ Corrected estimate, see Methods for details, *SE* Standard error, *NA* Not applicable, *Optimized GUI* Optimized graphical user interface, *ATS* Adaptive treatment strategy.

**Fig. 3:**
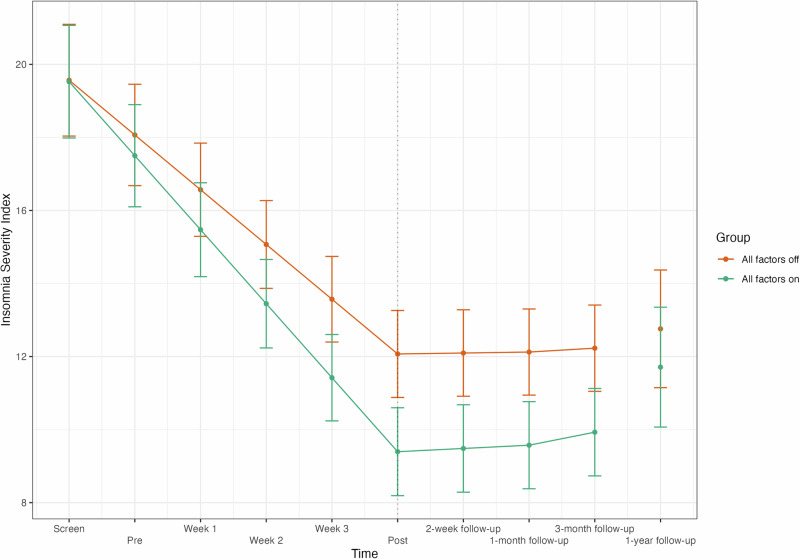
Insomnia symptom severity over time. Symptom severity over time for those who received all factors (‘All factors on’) compared to those who received none (‘All factors off’) as estimated by the multilevel model. Error bars indicate the 95% confidence interval of the estimates.

There was no statistically significant main effect of any factor. However, there was a statistically significant interaction effect of all three factors × the treatment period (Phase 1) on ISI. This additional effect over time further improved insomnia symptoms by 1.40 points, *d* = 0.26 (95% CI [0.10, 0.42]) on the ISI, leading to a predicted total improvement of 10.13 points (95% CI [9.79, 10.47]) on the ISI over the treatment period for those receiving all factors. In comparison, the predicted total improvement over the treatment period for those receiving no factors was 7.50 points (95% CI [7.16, 7.84]). Thus, participants receiving all factors improved an additional 2.63 points in comparison to those receiving no factors, *d* = 0.50 (95% CI [0.01, 0.99]), see Fig. 3.

Additionally, there was a statistically significant interaction effect of the optimized GUI × automated reminders × the follow-up period (Phase 2). This added effect over time further improved insomnia symptoms by 1.02 points on the ISI, *d* = 0.19 (95% CI [0.00, 0.34]). However, due to the other non-significant interaction effects with Phase 2, the predicted total improvement was 0.66 points (95% CI [0.24, 1.07]) on the ISI over the follow-up period for those receiving both the optimized GUI and automated reminders, *d* = 0.12 (95% CI [0.05, 0.20]).

The average total EQ score and ISI score were statistically significantly correlated, with higher engagement associated with lower insomnia symptoms, at post (r(428) = −0.40, *p* < 0.0001), three-month follow-up (r(405) = −0.35, *p* < 0.0001), and one-year follow-up (r(388) = −0.29, *p* < 0.0001).

### Usability, credibility, and treatment satisfaction

No main effect of the factors was observed on the CEQ. For the SUS, the optimized GUI showed a statistically significant main effect, with higher usability scores (*d* = 0.41, 95% CI [0.21, 0.62]). On the CSQ-8, the adaptive treatment strategy demonstrated a statistically significant main effect, resulting in higher satisfaction scores (*d* = 0.37, 95% CI [0.18, 0.56]). Further details are provided in Supplementary Table 2.

### Clinician time

Clinicians spent an average of 14.86 min (SD 5.24, range 6.5-65) per participant on the initial telephone interview (*n* = 447), and an average of 5.56 min (SD 3.04, range 1–28) per participant on the follow-up interview (*n* = 397). The optimized GUI and automated reminders did not require any clinician time.

Within the adaptive treatment strategy, the standardized procedure to assess risk of not sufficiently benefiting from treatment (*n* = 223) required two minutes per participant. Clinicians spent an average of 11.74 min (SD 10.43, range 0–49) per participant providing individualized support to those identified as at risk (*n* = 123). This resulted in a total of 13.74 min (SD 10.43, range 2–51) spent on the adaptive treatment strategy, including assessment and individualized support, per participant identified as at risk.

### Negative effects

A total of 89 (20%) participants reported a negative effect related to the intervention, most commonly tiredness or sleepiness (*n* = 57, 13%), negative impact on sleep (*n* = 15, 3%), and stress (*n* = 10, 2%). No serious adverse events were reported. There were no statistically significant differences in number of reported negative effects between factors ($\chi$²(2) = 0.30, *p* = 0.8588)

## Discussion

This factorial randomized controlled trial aimed to examine the impact of three intervention features – an optimized GUI, automated reminders, and an adaptive treatment strategy – on engagement and outcomes in a digital self-help intervention for insomnia incorporating the CBT-I components of sleep restriction and stimulus control. The average within-group effects on insomnia symptoms were large and comparable with those observed in previous studies on guided CBT-I, both post-treatment and at long-term follow-up^23^.

The findings from this factorial trial indicate that both the optimized GUI and the automated reminders significantly enhanced engagement, and that all intervention features contributed to improvements in insomnia symptoms within this digital self-help intervention. Specifically, while the automated reminders increased engagement in terms of log-in frequency and the number of days with recorded sleep logs, the optimized GUI demonstrated a broader impact – enhancing all measured dimensions of engagement: perceived engagement, log-in frequency, and sleep log adherence.

These findings are consistent with prior research. In a randomized controlled trial conducted by our research group, we found that an optimized GUI, compared to a standard version, increased engagement with a digital problem-solving intervention^12^. Furthermore, enhanced interface design features have been associated with improvements in both engagement and treatment efficacy in digital psychological interventions^9,10^. This supports the theoretical framework suggesting that optimized design facilitates interaction with core components of an intervention^24^, a factor that may be especially critical in self-help formats where no clinician is present to support participants through the content and exercises.

In addition, several systematic reviews have demonstrated that various types of reminders are positively associated with engagement^13^ and treatment outcomes^15,16^ in digital psychological interventions. However, a more recent review did not confirm this link with treatment outcomes^25^. The theoretical rationale for using prompts is based on the idea that individuals are more likely to engage with intervention-related tasks when reminders are delivered at appropriately timed moments^26^, highlighting the importance of tailoring reminders to the specific context and structure of the intervention. These mixed results may therefore reflect a mismatch between the reminders and the individual needs of patients or users – an aspect that has been emphasized as critical in intervention design^27^. Reminders may play a particularly important role in self-help interventions, as they can potentially compensate for the absence of clinician-provided prompts that typically encourage engagement.

There was no main effect of the adaptive treatment strategy on any of the engagement measures. However, the adaptive treatment strategy significantly increased treatment satisfaction, indicating that participants valued the support they received. It should be noted that the adaptive treatment strategy was introduced partway through the intervention, limiting its influence over the full four-week period. Previous studies emphasize the importance of sufficient time after risk prediction to support those unlikely to benefit^28^. For instance, in a 9-week intervention, prediction occurred at the third treatment week^19^, allowing support to be given over several weeks. In the brief format of the current intervention, the limited duration may have reduced the impact of the strategy. A slightly longer protocol may be needed for such an approach to influence engagement or outcomes meaningfully.

The combination of all three features was associated with an increased reduction in insomnia symptoms over the treatment period. More specifically, participants who received all three intervention components improved their ISI scores by nearly three *additional* points on average. This symptom change may be considered clinically important, as it represents approximately half of the improvement typically used to define a clinically meaningful improvement^29^.

The effects of the factors should be considered in relation to their associated costs. The addition of an optimized GUI and automated reminders required no clinician time, thus incurring no extra running cost for the healthcare provider. However, it is important to note that the development of new features, as well as the maintenance of systems, is associated with costs. The adaptive treatment strategy in this trial required an average of approximately 14 min of clinician time per at-risk participant over the four-week intervention period. This is quite low compared to previous research. In one trial evaluating an adaptive treatment strategy, clinicians spent an average of 14 additional minutes per week per at-risk participant during the remaining treatment period, resulting in a total of 70 additional minutes on average per at-risk participant^19^. Such running costs may be justified by the enhanced improvements in insomnia symptoms observed at the group level.

This study has several strengths and limitations. Its large sample size and low data attrition contribute to the robustness of the findings, while the factorial design allowed efficient comparisons between features. However, the study design lacked a comparison to another intervention, and the sample was 87% female – higher than typical, even considering general trends. The sample was also recruited via media outreach, which limits the generalizability to patients in routine care. Nevertheless, the sample showed a high clinical need, reflected by insomnia severity, a high proportion of comorbid conditions, and an average of 17 years of sleep difficulties.

This growing understanding of intervention design in the context of digital self-help – specifically, how to optimize engagement and treatment outcomes with minimal clinician time – may contribute to making effective treatment more accessible to individuals in need. While design features such as an optimized GUI and automated reminders can be implemented without requiring clinician time, the adaptive treatment strategy does involve some degree of clinician input. However, it is encouraging that in this trial, the adaptive strategy required only an average of 14 min of clinician time per at-risk participant.

Before implementing design features into digital interventions, it is essential to conduct trials that replicate the findings within the intended context of delivery – such as routine healthcare settings – to ensure their effectiveness and applicability in real-world conditions, where engagement is often lower^4,30^. Additionally, studies that identify which individuals are most likely to benefit from an adaptive treatment strategy would be valuable. The field would also benefit from further research investigating the causal relationship between engagement and outcomes.

In summary, the findings from this study suggest that using an optimized GUI and automated reminders can enhance engagement in digital self-help interventions for insomnia, and that incorporating both features, along with an adaptive treatment strategy, can improve symptom outcomes.

## Methods

### Study design

The study was a 2 x 2 x 2 single-blind factorial randomized controlled trial conducted in Sweden. The factorial design enables the calculation of main effects and interaction effects of factors in a resource-efficient way^31^. The study was approved by the Swedish Ethical Review Authority (ID: 2022-07226-01).

The study adheres to the CONSORT guidelines for reporting randomized controlled trials. Where the CONSORT 2010 guidelines were not directly applicable, we have followed the extended CONSORT 2010 Statement for reporting factorial randomized trials. This also applies to the construction of the participant flow diagram. See Supplementary Note 1 for a completed CONSORT checklist.

### Participants

Participants were recruited in Sweden via Facebook advertisements during two periods in 2023: between May 1st and June 5th, and between September 11th and October 23rd. All participants provided written informed consent at intake.

Inclusion criteria were: ≥ 18 years; ≥ 15 points on the Insomnia Severity Index (ISI)^32^; insomnia diagnosis according to the Diagnostic and Statistical Manual of Mental Disorders-5; proficiency in Swedish; no practical barriers to participation; access to a digital device connected to the Internet; and access to a mobile phone.

Exclusion criteria were: untreated sleep disorder (other than insomnia) that required other care; somatic or psychiatric illness considered a contraindication for the treatment (e.g., bipolar type I, psychosis) or requiring other acute treatment (other somatic/psychiatric comorbidity was allowed); alcohol/drug abuse or medication with substantial side effects on sleep (sleep medication was allowed); and working night shifts.

### Randomization and masking

The factors manipulated in the trial were an optimized GUI (factor 1), automated reminders (factor 2) and an adaptive treatment strategy (factor 3). Participants were randomized (1:1:1:1:1:1:1:1) to use a digital self-help intervention for insomnia with none of the factors, one of the factors, or a combination of the factors. This created eight equally large randomization groups, which corresponded to three dichotomous intervention features set to on or off, see Fig. 1.

The randomization procedure was handled by an administrator blinded to the conditions, using an R script to allocate participants to the eight groups. The administrator generated the randomization sequence before data collection. A blocking method was used, with a size of either 40 or 56. Upon randomization request from the study coordinator (AH), the administrator used the R script to allocate anonymized IDs to numbers (1–8) corresponding to the eight groups, which were masked to the administrator. The anonymized ID was later sent together with a randomization number to the study coordinator who was responsible for both enrollment of participants and assignment of participants to trial groups. Participants did not receive information about the trial evaluating different factors, nor about the factors, and thus were blinded to what conditions were manipulated in the trial, and what condition they were allocated to.

### Procedures

Before inclusion, all participants completed ISI, as a baseline assessment of insomnia symptoms. The ISI has a score range of 0 to 28, with lower scores indicating better outcomes. Scores are categorized as follows: 0–7 indicates no insomnia, 8–14 subthreshold insomnia, 15–21 moderate insomnia, and 22–28 severe insomnia. Each participant was then contacted per telephone for an assessment interview. Following inclusion, participants completed baseline assessments for depression and anxiety using the Patient Health Questionnaire-9 (PHQ-9)^33^, score range 0–27, with lower scores indicating better outcomes, and the Generalized Anxiety Disorder-7 (GAD-7)^34^, score range 0–21, with lower scores indicating better outcomes. Participants then accessed a four-week digital self-help intervention for insomnia via a secure online platform. After the treatment period, all participants were contacted by telephone for a brief follow-up interview.

### Intervention and factors

The core version of the intervention was developed for the previously mentioned feasibility trial of FastAsleep^22^, and was designed from the outset to function as a self-help format. It incorporated the CBT-I components of sleep restriction and stimulus control, which correspond to brief behavioral treatment for insomnia^35^.

At the beginning of the treatment, participants set a sleep window (i.e., the time frame that the participant were allowed to spend in bed each night) based on their habitual sleep time, and were encouraged to implement it immediately. The goal was to consolidate sleep and improve sleep efficiency, i.e., the proportion of time spent asleep while in bed. Participants adjusted the sleep window based on sleep efficiency ( > 85%) and daytime sleepiness, and kept a sleep diary to track total sleep time, wakefulness, and time in bed, which was used to calculate sleep efficiency.

Depending on their randomization group, participants got to use the intervention with zero or more of the three factors manipulated in the trial.

Factor 1 was an optimized GUI, co-designed with user experience specialists to ensure simplicity and intuitiveness. Participants not assigned to this factor used a basic GUI similar to one employed in Swedish healthcare for over a decade. See Supplementary Table 3 for GUI comparisons. Factor 2 was daily automated reminders, sent at 9 a.m. via text messages, prompting participants to log their sleep in the diary (e.g., “Hi [name]! Please remember to register your sleep: [hyperlink to treatment platform]. Best regards, the Sleep intervention”). Participants not assigned to this factor did not receive these reminders.

Factor 3 was an adaptive treatment strategy comprising an assessment regarding whether participants classified as at risk of not sufficiently benefiting from treatment, and individualized support to those identified as such. The author AH evaluated participants based on three predefined criteria related to engagement and early change that have been found to be associated with treatment outcome^36,37^: 1) whether participants set a sleep window within the first five days, 2) whether participants logged their sleep at least 50% of the days over the first two weeks, and 3) whether participants achieved at least 25% symptom improvement over the first two weeks. Participants failing any criteria were classified as at risk. The criteria were also based on the weekly symptom progression previously observed in the feasibility trial of FastAsleep, where patients demonstrated a 33% improvement on average in ISI scores within the first two weeks^22^. The support, provided by clinical psychologists (authors AH and AR), included a semi-structured phone assessment and ongoing assistance via phone or platform messages, focusing on motivation, coping strategies and problem-solving.

### Outcomes

The primary outcome was treatment engagement, measured using a study-specific Engagement Questionnaire (EQ), see Supplementary Note 2. The EQ included three items scored from 0 to 7, with total scores ranging from 0 to 21, reflecting the number of days participants engaged with the core techniques over the past week. Thus, higher scores indicate greater engagement. The EQ was assessed weekly during the intervention, and at two weeks, one month, three months, and one year after treatment. The EQ scores were averaged per week across three periods: the treatment period (week 1 to post), the short-term follow-up period (post to three-month follow-up), and at one-year follow-up. Follow-up assessments were conducted to examine whether participants continued applying the core techniques over time.

The secondary outcomes included objective engagement measures, insomnia symptoms, system usability, treatment credibility, treatment satisfaction, clinician time, and any negative effects of the treatment.

Objective engagement data included the number of days with recorded sleep logs (up to 27 days) during treatment and the number of logins to the treatment platform. Insomnia symptoms were assessed using the ISI^32^ at baseline, pre-treatment, weekly during the intervention, and after treatment at two weeks, one month, three months, and one year.

System usability was evaluated at mid-treatment using the System Usability Scale (SUS)^38^, a self-rated questionnaire with scores ranging from 0 to 100, where higher scores indicate better usability. Treatment credibility was also assessed at mid-treatment using a five-item version of the Credibility/Expectancy Questionnaire (CEQ)^39^, which has a score range of 0 to 50, with higher scores reflecting greater credibility. Treatment satisfaction was measured at post-treatment using the Client Satisfaction Questionnaire-8 (CSQ-8)^40^, with scores ranging from 8 to 32, where higher scores denote higher satisfaction. Clinician time was continuously logged on the intervention platform. After completing the treatment, participants reported any negative effects.

### Statistical analyses

The sample size was determined through power calculations for the primary outcome analyses, with 80% power and Cohen’s *d* = 0.30, considered a reasonable threshold for distinguishing relevant main effects of each factor. An additional sample size of 10% was planned for recruitment to account for potential missing data, resulting in a desired number of at least 385 participants.

To investigate the effects of the factors on ISI, multilevel regression analysis was conducted^41^ incorporating a random intercept and random slope for time, with the intercept set at the post-treatment timepoint. If the model failed to converge, the correlation between the random slope and intercept was dropped for simplification. For EQ and the objective engagement measures, SUS, CEQ and CSQ-8, linear regression was utilized. Pearson’s correlation coefficient was calculated between the number of days with recorded sleep logs and EQ to check the consistency between these measures, as well as between EQ and ISI to examine their relationship. Standardized effect sizes (*d*) for the mixed models were calculated based on the models’ coefficients^42^, whereas the others were calculated as Cohen’s *d* using the pooled standard deviation.

In all regression models, the factors and their interactions served as predictors, with factors effect coded −0.50 = factor off and 0.50 = factor on, to enhance power for higher-order interactions^31^. This allows the main effects of factors to be interpreted as the mean effect of each factor, but higher-order interactions must be adjusted by 0.50 for two-way interactions and 0.25 for three-way interactions (denoted as *b*_*corr*_ in the text)^43^.

When analyzing ISI, two timepieces, coded for separate slopes, and their interactions with the factors were included in the regression model: one (Phase 1) for the treatment period (baseline to post-treatment) and another (Phase 2) for the follow-up period (post-treatment to one-year follow-up). For Phase 1, ISI was measured six times over six weeks. For Phase 2, ISI was measured four times over 52 weeks. Time was coded in weeks for all regression models, beginning at week zero.

An intention-to-treat analysis (ITT) was employed for ISI, EQ and the objective engagement measures. Data management and analysis were performed using R, version 4.2.0, using lme4 for mixed models^44^. The study was pre-registered 2023-04-11 on ClinicalTrials.gov (ID: NCT05826002).

## Supplementary information

Supplementary Materia

## Data Availability

Deidentified participant data will be made available on reasonable request to the corresponding author. Due to participant integrity, the data cannot be made publicly available.

## Code availability

Analyses were conducted using R, version 4.2.0, using lme4 for mixed models^44^. Code will be made available upon reasonable request.
