# Supplementary material for: Effects of intervention design on engagement and outcomes in digital self-help for insomnia – factorial RCT
Source: NPJ Digit Med. 2025 Jul 8;8:416. doi: 10.1038/s41746-025-01839-0 (PMC12238518; doi:10.1038/s41746-025-01839-0)
Supplement: Supplementary file 1 — Supplementary Materia [file 41746_2025_1839_MOESM1_ESM.pdf]

Supplementary Note 1

CONSORT 2010 checklist of information to include when reporting a randomised trial*

Section/Topic	Item No	Checklist item	Reported on page No
**Title and abstract**			
	1a	Identification as a randomised trial in the title	1
	1b	Structured summary of trial design, methods, results, and conclusions (for specific guidance see CONSORT for abstracts)	1
**Introduction**			
Background and objectives	2a	Scientific background and explanation of rationale	2-3
	2b	Specific objectives or hypotheses	3
**Methods**			
Trial design	3a	Description of trial design (such as parallel, factorial) including allocation ratio	7-8
	3b	Important changes to methods after trial commencement (such as eligibility criteria), with reasons	NA
Participants	4a	Eligibility criteria for participants	7
	4b	Settings and locations where the data were collected	7
Interventions	5	The interventions for each group with sufficient details to allow replication, including how and when they were actually administered	8-9
Outcomes	6a	Completely defined pre-specified primary and secondary outcome measures, including how and when they were assessed	9
	6b	Any changes to trial outcomes after the trial commenced, with reasons	NA
Sample size	7a	How sample size was determined	9
	7b	When applicable, explanation of any interim analyses and stopping guidelines	NA
Randomisation:			
Sequence generation	8a	Method used to generate the random allocation sequence	8
	8b	Type of randomisation; details of any restriction (such as blocking and block size)	8
Allocation concealment mechanism	9	Mechanism used to implement the random allocation sequence (such as sequentially numbered containers), describing any steps taken to conceal the sequence until interventions were assigned	8
Implementation	10	Who generated the random allocation sequence, who enrolled participants, and who assigned participants to interventions	8
Blinding	11a	If done, who was blinded after assignment to interventions (for example, participants, care providers, those	8

		assessing outcomes) and how	
	11b	If relevant, description of the similarity of interventions	8-9
Statistical methods	12a	Statistical methods used to compare groups for primary and secondary outcomes	10
	12b	Methods for additional analyses, such as subgroup analyses and adjusted analyses	10
**Results**			
Participant flow (a diagram is strongly recommended)	13a	For each group, the numbers of participants who were randomly assigned, received intended treatment, and were analysed for the primary outcome	Figure 1
	13b	For each group, losses and exclusions after randomisation, together with reasons	Figure 1
Recruitment	14a	Dates defining the periods of recruitment and follow-up	7
	14b	Why the trial ended or was stopped	9
Baseline data	15	A table showing baseline demographic and clinical characteristics for each group	Table 1
Numbers analysed	16	For each group, number of participants (denominator) included in each analysis and whether the analysis was by original assigned groups	3-5
Outcomes and estimation	17a	For each primary and secondary outcome, results for each group, and the estimated effect size and its precision (such as 95% confidence interval)	3-5
	17b	For binary outcomes, presentation of both absolute and relative effect sizes is recommended	NA
Ancillary analyses	18	Results of any other analyses performed, including subgroup analyses and adjusted analyses, distinguishing pre-specified from exploratory	NA
Harms	19	All important harms or unintended effects in each group (for specific guidance see CONSORT for harms)	5
**Discussion**			
Limitations	20	Trial limitations, addressing sources of potential bias, imprecision, and, if relevant, multiplicity of analyses	6
Generalisability	21	Generalisability (external validity, applicability) of the trial findings	6
Interpretation	22	Interpretation consistent with results, balancing benefits and harms, and considering other relevant evidence	7
**Other information**			
Registration	23	Registration number and name of trial registry	1, 10
Protocol	24	Where the full trial protocol can be accessed, if available	NA
Funding	25	Sources of funding and other support (such as supply of drugs), role of funders	1, 10

Citation: Schulz KF, Altman DG, Moher D, for the CONSORT Group. CONSORT 2010 Statement: updated guidelines for reporting parallel group randomised trials. BMC Medicine. 2010;8:18.
© 2010 Schulz et al. This is an Open Access article distributed under the terms of the Creative Commons Attribution License (http://creativecommons.org/licenses/by/2.0), which permits unrestricted use, distribution, and reproduction in any medium, provided the original work is properly cited.

*We strongly recommend reading this statement in conjunction with the CONSORT 2010 Explanation and Elaboration for important clarifications on all the items. If relevant, we also recommend reading CONSORT extensions for cluster randomised trials, non-inferiority and equivalence trials, non-pharmacological treatments, herbal interventions, and pragmatic trials. Additional extensions are forthcoming: for those and for up-to-date references relevant to this checklist, see www.consort-statement.org.

Supplementary Note 2 Engagement Questionnaire (EQ)

Below you find three questions concerning your work and engagement with the sleep program during the past week.

1. During the past week, how many days have you worked on following the overall sleep recommendations?

( ) Not at all
( ) 1 day
( ) 2 days
( ) 3 days
( ) 4 days
( ) 5 days
( ) 6 days
( ) 7 days

2. In the program, you're advised to sleep only within your designated sleep window. During the past week, how many days have you focused on doing that?

( ) Not at all
( ) 1 day
( ) 2 days
( ) 3 days
( ) 4 days
( ) 5 days
( ) 6 days
( ) 7 days

3. In the program, you're advised to leave your bed if you've been awake for more than 20 minutes. During the past week, how many days did you stay in bed for longer than 20 minutes while awake? *The scoring for this item was reversed in the sum.*

( ) Not at all
( ) 1 day
( ) 2 days
( ) 3 days
( ) 4 days
( ) 5 days
( ) 6 days
( ) 7 days

Supplementary Table 1 Effects of the factors on the number of days with recorded sleep logs and number of logins during the intervention period

*Statistically significant effect; CI = Confidence interval; b_{corr} = Corrected estimate, see Methods for details; SE = Standard error; NA = Not applicable; Optimized GUI = Optimized graphical user interface; ATS = Adaptive treatment strategy.

	Days with recorded sleep logs				Number of logins			
	**Estimate [95% CI]**	**b_{corr} [95% CI]**	**SE**	**p**	**Estimate [95% CI]**	**b_{corr} [95% CI]**	**SE**	**p**
**Intercept**	19.33 [18.39, 20.28]	NA	0.48	<.0001*	22.07 [20.95, 23.19]	NA	0.57	<.0001*
**Optimized GUI**	4.58 [2.69, 6.47]	4.58 [2.69, 6.47]	0.96	<.0001*	3.37 [1.13, 5.61]	3.37 [1.13, 5.61]	1.14	.0033*
**Automated reminders**	2.70 [0.80, 4.59]	2.70 [0.80, 4.59]	0.96	.0054*	4.00 [1.76, 6.24]	4.00 [1.76, 6.24]	1.14	.0005*
**ATS**	0.88 [-1.01, 2.77]	0.88 [-1.01, 2.77]	0.96	.3600	0.83 [-1.42, 3.07]	0.83 [-1.42, 3.07]	1.14	.4697
**Optimized GUI×Automated reminders**	1.64 [-2.15, 5.42]	0.82 [-1.08, 2.71]	1.93	.3950	4.49 [0.01, 8.97]	2.25 [0.01, 4.49]	2.28	.0496*
**Optimized GUI×ATS**	1.69 [-2.09, 5.48]	0.85 [-1.05, 2.74]	1.93	.3798	0.70 [-3.78, 5.19]	0.35 [-1.89, 2.6]	2.28	.7579
**Automated reminders×ATS**	-1.18 [-4.97, 2.60]	-0.59 [-2.49, 1.30]	1.93	.5396	-1.28 [-5.76, 3.20]	-0.64 [-2.88, 1.6]	2.28	.5752
**Optimized GUI×Automated reminders×ATS**	-2.79 [-10.36, 4.78]	-0.7 [-2.59, 1.20]	3.85	.4687	-2.95 [-11.91, 6.01]	-0.74 [-2.98, 1.5]	4.56	.5180

Supplementary Table 2 Effects of the factors on the System Usability Scale, Credibility/Expectancy Questionnaire and the Client Satisfaction Questionnaire-8

*Statistically significant effect; CI = Confidence interval; b_{corr} = Corrected estimate, see Methods for details; SE = Standard error; NA = Not applicable; Optimized GUI = Optimized graphical user interface; ATS = Adaptive treatment strategy.

	**System Usability Scale**				**Credibility/Expectancy Questionnaire**				**Client Satisfaction Questionnaire-8**			
	**Estimate [95% CI]**	**b_{corr} [95% CI]**	**SE**	**p**	**Estimate [95% CI]**	**b_{corr} [95% CI]**	**SE**	**p**	**Estimate [95% CI]**	**b_{corr} [95% CI]**	**SE**	**p**
**Intercept**	73.77 [71.92, 75.62]	NA	0.94	<.0001*	33.26 [32.20, 34.31]	NA	.54	.0000*	22.70 [22.21, 23.20]	NA	.25	.0000*
**Optimized GUI**	7.43 [3.74, 11.13]	7.43 [3.74, 11.13]	1.88	.0001*	0.69 [-1.42, 2.81]	0.69 [-1.42, 2.81]	1.07	.5188	0.65 [-0.34, 1.64]	0.65 [-0.34, 1.64]	0.50	.1956
**Automated reminders**	-0.11 [-3.81, 3.59]	-0.11 [-3.81, 3.59]	1.88	.9539	-0.46 [-2.57, 1.65]	-0.46 [-2.57, 1.65]	1.07	.6702	0.03 [-0.95, 1.02]	0.03 [-0.95, 1.02]	0.50	.9453
**ATS**	-0.35 [-4.05, 3.34]	-0.35 [-4.05, 3.34]	1.88	.8511	1.17 [-0.94 3.29]	1.17 [-0.94 3.29]	1.07	.2752	1.90 [0.91, 2.89]	1.90 [0.91, 2.89]	0.50	.0002*
**Optimized GUI ×Automated reminders**	-6.68 [-14.07, 0.72]	-3.34 [-7.04, 0.36]	3.76	.0766	-0.66 [-4.88, 3.57]	-0.33 [-2.44, 1.79]	2.15	.7602	1.26 [-0.71, 3.24]	0.63 [-0.36, 1.62]	1.00	.2091
**Optimized GUI×ATS**	1.07 [-6.32, 8.47]	0.54 [-3.16, 4.24]	3.76	.7754	0.51 [-3.71, 4.74]	0.26 [-1.86, 2.37]	2.15	.8117	0.50 [-1.47, 2.48]	0.25 [-0.74, 1.24]	1.00	.6159
**Automated reminders ×ATS**	3.63 [-3.77, 11.02]	1.82 [-1.88, 5.51]	3.76	.3355	3.71 [-0.52, 7.93]	1.86 [-0.26, 3.97]	2.15	.0852	0.03 [-1.94, 2.01]	0.02 [-0.97, 1.01]	1.00	.9725
**Optimized GUI ×Automated reminders ×ATS**	-8.69 [-23.48, 6.10]	-2.17 [-5.87, 1.53]	7.52	.2486	-4.05 [-12.50, 4.40]	-1.01 [-3.13, 1.10]	4.30	.3460	1.21 [-2.74, 5.15]	0.30 [-0.69, 1.29]	2.01	.5484

Supplementary Table 3 Differences Between Graphical User Interfaces

	Optimized Graphical User Interface	
	**On**	**Off**
**Type of responsive design**	Mobile-first	Desktop-first
**Division of content**	Subsections based on treatment week and type of content, pages and expandable learn-more options	Subsections based on treatment week
**Presentation of psychoeducation**	Presented in small chunks	Presented in scrollable pages
**Sleep registration and sleep window adjustment tool**	Stepwise fashion	Digital worksheet
**Instructions**	Separate instructions at each step	All instructions next to the digital worksheet
**Examples and suggestions**	Displayed within expandable containers	Displayed as part of the rest of the content
**Automated features**	Information entered by participant in previous steps of the intervention automatically synched to upcoming steps when relevant; Participant automatically moved to the relevant step of the intervention based on previous activity	None
